# Investigatory pathway and principles of patient selection for epilepsy surgery candidates: a systematic review

**DOI:** 10.1186/s12883-020-01680-w

**Published:** 2020-03-17

**Authors:** Arash Ghaffari-Rafi, Jose Leon-Rojas

**Affiliations:** 1 University of Hawai’i at Mānoa, John A. Burns School of Medicine, Honolulu, Hawaii USA; 2 University College London, Queen Square Institute of Neurology, London, England, UK; 3 Universidad Internacional del Ecuador, Medical School, Quito, Ecuador

**Keywords:** Patient selection, Epilepsy surgery, Temporal lobe epilepsy, Seizure, Seizure freedom, Drug resistant epilepsy

## Abstract

**Background:**

The predominant treatment for epilepsy is pharmacotherapy, yet 20–40% do not respond to anti-epileptic drugs. After becoming pharmacoresistant, some patients are worked-up to determine candidacy for epilepsy surgery. Despite the 2009 American Epilepsy Society guidelines, there is no broadly accepted criteria for the investigatory pathway and principles of patient selection for epilepsy surgery candidates. The objective of this systematic review is to elucidate what diagnostic pathways clinicians globally utilize.

**Methods:**

Utilizing the Preferred Reporting Items for Systematic Reviews and Meta-Analysis (PRISMA) and the Cochrane Handbook of Systemic Reviews of Interventions, we conducted a systematic review through MEDLINE, Embase, and CENTRAL.

**Results:**

From 2092 screened articles, 14 met inclusion criteria for qualitative synthesis. Structural MRI was required in all investigatory pathways. All but two articles required neuropsychological assessment. Six required neuropsychiatric assessment. Two protocols mentioned assessing the patient’s support network. Three other protocols mentioned discussing expectations with patients. One also motioned conducing an occupational evaluation and making all surgery decisions in a multidisciplinary management conference. fMRI and the Wada test were required assessments in seven of the protocols. [18F]FDG-PET and SPECT were ancillary for all but three articles (where they were required). MEG and intracranial EEG were only mentioned as ancillary. Magnetic resonance (MR) spectroscopy was required at two institutes. With regards to the actual indication for selecting patients to begin the investigatory pathway, seven of the articles used a variation of the International League Against Epilepsy definition of refectory epilepsy, while one incorporated patient social history.

**Conclusions:**

Despite attempts to standardize patient selection and investigatory pathways, no two protocols were identical. Scalp video/EEG telemetry, structural MRI, and neuropsychological assessment were the only assessments utilized in nearly all protocols. Socioeconomic restrictions appear to play a role in determining which tests are utilized in the investigatory pathway—not just for developing countries. However, cost-effective assessments, such as assessing patient support network and providing realistic expectation of outcomes, were only utilized in few protocols. In addition, no advanced imaging technologies (i.e., qMRI, 3D-MMI) were utilized. Overall, even amongst expert examiners there is significant variation throughout epilepsy centers globally, in selecting candidates and working up patients.

## Background

Epilepsy impacts over fifty million globally, with an annual incidence of two-million [1]. The predominant treatment is pharmacotherapy with anti-epileptic drugs (AED), yet 20–40% do not respond to AEDs [2, 3]. These AED unresponsive patients are deemed pharmacoresistant after failing to respond to a predetermined trial of AEDs [4]. Although there is a lack of consensus on the definition of pharmacoresistance, the International League Against Epilepsy (ILAE) Commission on Therapeutic Strategies, defined pharmacoresistance as the failure to achieve seizure freedom after utilization of at least two appropriately dosed first-line AEDs [5]. Typically, after becoming pharmacoresistant, some patients are then worked-up to determine candidacy for epilepsy surgery [6]. Epilepsy surgery is particularly promising, especially after the first randomized controlled trial for temporal lobe epilepsy (TLE) found surgery resulted in a greater proportion of patients becoming seizure free—current data demonstrates 60–80% of postoperative patients become seizure free [7–9].

Depending on the epilepsy type, there are several surgical methods, and each surgical type utilized will vary slightly with regards to the goals of presurgical evaluation [6]. For instance, patients with TLE can either undergo standard or tailored anteromesial resection [10]. Those undergoing standard resection primarily require identification of the epileptogenic zone boundaries, while for tailored surgery preoperative evaluation must also pinpoint the neocortex (primary motor and language cortical areas) [6]. Likewise, neocortical epilepsy patients consistently require a tailored approach, hence preoperative assessment involves defining the exact epileptogenic tissue [6, 11]. On the other hand, hemispherectomy and hemispherotomy are standardized techniques, where the goals of the preoperative assessment seek to confirm whether the contralateral hemisphere can maintain adequate function and the ipsilateral hemisphere has no remnant non-epileptogenic tissue with important functions [6, 12]. Lastly, corpus callosotomy preoperative assessment involves validation that a more localized operation cannot be performed [6]. Despite these generalizations on presurgical assessment, the specifics of patient workup are relatively inconsistent between institutes [13].

In 2009, a roadmap was provided for the investigatory pathway of epilepsy surgery, by the consensus conference of the American Epilepsy Society [14]. Set forth was a multidisciplinary methodological approach involving: clinical history, physical exam, scalp video/electroencephalogram (EEG) telemetry, structural magnetic resonance imaging (MRI; epilepsy protocol), neuropsychological assessment, neuropsychiatric assessment, social work and nursing assessment of patient support network, and consulting the patient on realistic expectations of outcomes [14]. Additionally, mentioned ancillary tests included: functional MRI (fMRI), the Wada test, fluorodeoxyglucose F18 positron emission tomography ([18F]FDG-PET), ictal single photon emission computed tomography (SPECT), magnetoencephalography (MEG), and intracranial EEG electrodes [14].

With regards to these presurgical investigations, they all play a vital role in selecting patients for epilepsy surgery. For instance, video-EEG records scalp EEG activity while concurrently video recording the patient for the purpose of producing an electroclinical correlation regarding the seizure [15]. Meanwhile, MRI seeks to provide structural identification of the epileptogenic zone, particularly in those cases of a localized abnormality such as malformations of cortical development [16, 17]. In addition to providing a qualitative assessment (i.e., structural analysis), MRI can also be used to provide a quantitative analysis (quantitative MRI [qMRI]) of signal intensity, which through computational methods and machine learning uncovers epileptogenic foci that are not accurately identified by expert human examiners [18, 19]. qMRI has been used to automatically classify and diagnose the laterality of TLE as well as via T2-relaxometry can automatically identify hippocampal sclerosis [20, 21]. fMRI is used to assess the impact of epileptic activity on various physiologic tasks (memory, language, etc.) [4].

The neuropsychological evaluation seeks to determine the brain region impacted, the potential for postoperative memory loss, and hemisphere dominance (i.e., Wada test) [4, 22, 23]. Neuropsychiatric assessment determines comorbid psychiatric disorders—which are more likely with epilepsy; psychiatric disorders increase risk of suicide and can be surgery contraindications [24, 25]. Lastly, ancillary tests (tests generally reserved for difficult cases where the epileptogenic zone cannot be clearly defined), such as SPECT, PET, MEG, and intracranial EEG are utilized to better define dubious epileptogenic zones [26–28]. Although, intracranial EEG (such as steroelectroencephalography [SEEG]) is useful in identifying epileptogenic zones not easily discernably via non-invasive testing, it is often deemed ancillary due to the added risk to patients conferred by intracranial electrodes. One study examining 242 epilepsy patients associated SEEG with a 23% complication rate and 9% surgical revision rate [29].

In combination, these tests provide valuable insight into determining the appropriate surgical candidates. In addition to providing diagnostic information vital for classification of the surgical candidates, all these imaging techniques are also useful for surgical planning and execution [30]. Particularly, different qualitative and quantitative MRI acquisitions, fMRI, EEG, and the aforementioned ancillary tests can be combined in order to create a three-dimensional multimodality image (3D-MMI) custom tailored to each specific patient [31]. This 3D-MMI can provide important information on the patient’s particular anatomy as well as the location of functional white matter tracts to be avoided during surgery; such therefore reduces surgical morbidity by increasing precision during surgery and resulting in improved post-operative seizure status [32, 33].

Overall, despite the 2009 American Epilepsy Society guidelines, there is no broadly accepted criteria for the investigatory pathway and principles of patient selection for epilepsy surgery candidates. Thus, the objective of this systematic review is to elucidate what diagnostic pathways clinicians globally utilize.

## Methods

The systematic review was conducted in accordance with the Preferred Reporting Items for Systematic Reviews and Meta-Analysis (PRISMA) and the Cochrane Handbook of Systematic Reviews of Interventions [34–36].

### Eligibility criteria

Only articles providing a detailed description of the institution’s protocol for investigating epilepsy surgery were included. Exclusion criteria included protocols that exclusively focused on the use of neurostimulators (i.e. vagal nerve stimulation), deep brain stimulation, or other interventional method (i.e., laser interstitial thermal therapy), as such are not technologies widely available in under-resourced or developing countries. Literature reviews were also excluded.

#### Participants

Studies including data for adult humans (18 years or older) undergoing non-palliative surgery preoperative investigations were included.

#### Language

Only English and Spanish language articles were included.

#### Information sources

Medical subheadings (MeSH) and text words related to epilepsy, seizure, and patients, were utilized for the search strategy. Medline (PubMed interface, 2009 onwards), Embase (Ovid interface, 2009 onwards), and Cochrane Central Register for Controlled Trials (CENTRAL; Wiley interface, current issue), were all searched. 1 January 2009 was selected as the start date for the search, based on the December 2009 American Epilepsy Society consensus conference recommending a diagnostic workup pathway [14].

#### Search strategy

Other than dates, no limits were utilized in the database search limitations. An electronic search examined Embase (January 1, 2009 to February 17, 2019), MEDLINE (January 1, 2009 to February 17, 2019), and CENTRAL (January 1, 2009 to June 13, 2019); the Additional file 1 provides the search protocols, including keywords. Specific search strategies were developed under guidance of Queen Square Institute of Neurology (IoN) library and statistical services staff with expertise in systemic review searches. The search was conducted independently by both authors (A.G.R. and J.R.L.); when in disagreement, the conflicting articles were discussed, and consensus was reached. To assess the search sensitivity and quality, robust target references were utilized—all of which were identified by the search protocols [4, 14, 37].

### Study records

#### Data management

Results of the literature search were imported to EndNote X9 (Clarivate Analytics, Philadelphia, Pennsylvania). Software utilization aimed to reduce data entry errors and reduce bias, such as by deduplicating references.

#### Selection process

The authors screened all titles and abstracts on the basis of the inclusion criteria. Subsequently, literature meeting inclusion criteria (including uncertain results) had the full-text reviewed. For results that met inclusion criteria, the literature was included in the systematic review.

#### Data items

In accordance with recommendations from the *Cochrane Handbook for Systematic Reviews of Interventions* (chapter 7), the following data was collected into a Microsoft Excel spreadsheet: author, publication year, journal citation; institution or epilepsy center location; patient selection methodology; diagnostic workup (i.e., clinical history, physical examination, scalp video/EEG telemetry, structural MRI, neuropsychological assessment, neuropsychiatric assessment, social care/nursing assessment [of support network], fMRI, Wada test, [18F]FDG-PET, ictal SPECT, MEG, intracranial EEG electrodes, providing patient realistic expectation of outcomes, MR spectroscopy, conducting a patient management conference, occupational evaluation) [34].

#### Data synthesis

From each identified protocol we collected the forms of assessments (i.e., structural MRI, neuropsychiatric assessment, etc.) conducted and whether each assessment was listed as required or ancillary. Data was then placed into tables allowing for relative comparison of patient selection criteria and diagnostic workup protocols. For each assessment, a proportion was determined to quantify the number of protocols that defined the assessment as required or ancillary.

## Results

**Fig. 1:**
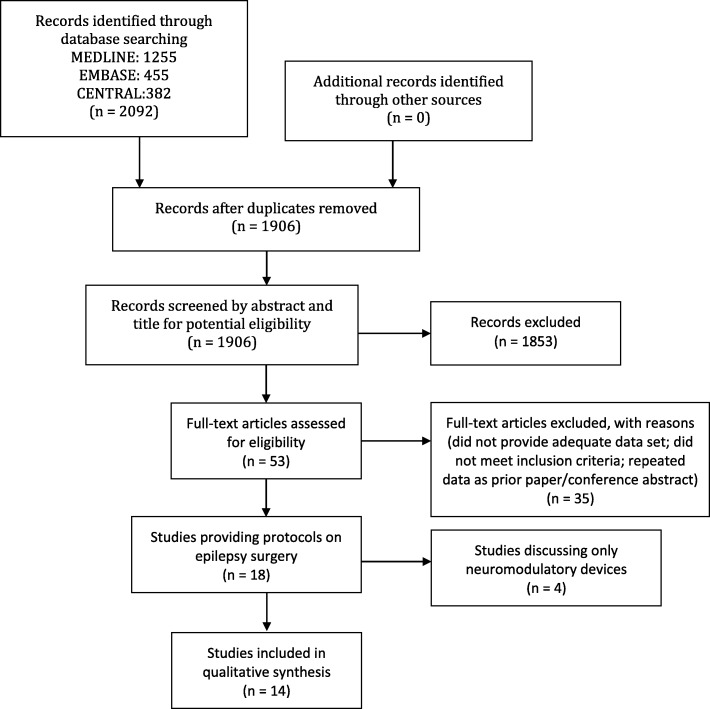
PRISMA Flow Diagram

MEDLINE, Embase, and CENTRAL identified 1255, 455, and 469 abstracts respectively, for a total of 2092; after deduplication, there were 1906 (Fig. 1). Eighteen articles met inclusion criteria, however four were excluded for only discussing neuromodulatory devices, thus leaving 14 articles for qualitative synthesis into Table 1; as one article was a survey, ultimately 13 protocols were included in the review.

**Table 1:** Investigatory Pathways. (✓) indicates requirement according to the article protocol. Note the National Temporal Lobectomy Survey [38] was not an individual protocol, hence was not included in the proportion calculations

Citation	Clinical History	Physical Examination	Scalp Video/EEG Telemetry	Structural MRI	Neuropsychological Assessment	Neuropsychiatric Assessment	Social Care/Nursing Assessment (of support network)	fMRI	Wada Test	[18F] FDG-PET	Ictal SPECT	MEG	Intracranial EEG Electrodes	Realistic Expectation of Outcomes	MR Spectroscopy	Patient Management Conference	Occupational Evaluation
London, England 2011 [14]	✓	✓	✓	✓	✓	✓	✓	Ancillary	Ancillary	Ancillary	Ancillary	Ancillary	Ancillary	✓			
National Temporal Lobectomy Survey; United States 2009 [39]					32–36.8%			28.6–36.8%	52–64.7% deemed essential	Considerable variation	Considerable variation	Considerable variation		nearly all inform of postoperative deficits			
Havana, Cuba 2009 [40]	✓	✓	✓	✓	✓	✓					✓				✓		
Gainesville, Florida 2009 [41]	✓		✓	✓						Ancillary	Ancillary	Ancillary	Ancillary				
Munich, Germany 2009 [42]	✓		✓	✓	✓			✓	✓	✓	✓						
Spanish Neurosurgical Society; Spain 2009 [43]	✓	✓	✓	✓	✓	✓	✓	✓	Ancillary	Ancillary	Ancillary	Ancillary	Ancillary	✓		✓	✓
Kerala, India 2010 [37]	✓	✓	✓	✓	✓	✓		✓	✓	✓	✓		Ancillary			✓	✓
Cleveland, Ohio 2010 [38]	✓		✓	✓	✓			✓		Ancillary	Ancillary						
Phoenix, Arizona 2011 [44]			✓	✓	✓				✓	Ancillary	Ancillary		Ancillary				
Oslo, Norway 2012 [45]	✓	✓	✓	✓	✓	✓			✓	Ancillary	Ancillary	Ancillary	Ancillary	✓	Ancillary		
Toronto, Canada 2012 [46]	✓	✓	✓	✓	✓			✓	✓	Ancillary	Ancillary	Ancillary					
Italian League Against Epilepsy; Italy 2013 [4]			✓	✓	✓	✓		✓		Ancillary	Ancillary		Ancillary				
Ankara, Turkey 2014 [47]			✓	✓		✓		✓	✓	✓			Ancillary		✓		
Japan 2016 [48]	✓		✓	✓	✓					Ancillary	Ancillary	Ancillary					
Listed as Required	10/13	6/13	13/13	13/13	11/13	7/13	2/13	7/13	6/13	3/13	3/13	0/13	0/13	3/13	2/13	2/13	2/13
Listed as Ancillary	0/13	0/13	0/13	0/13	0/13	0/13	0/13	1/13	2/13	9/13	9/13	6/13	8/13	0/13	1/13	0/13	0/13

Epilepsy surgery protocols were identified for: the National Hospital for Neurology and Neurosurgery in London, United Kingdom; the International Neurological Restoration Center in Cuba; the Madhavan Nayar Center for Comprehensive Epilepsy Care in India; the Spanish Neurosurgical Society in Spain; the Italian League Against Epilepsy; National Center Hospital for Neurology and Psychiatry in Japan; Barrows Neurological Institute in Arizona; Gazi University in Turkey; Toronto Western Hospital in Canada; Oslo University Hospital in Norway; University of Munich in Germany; University of Florida in the United States; Cleveland Clinic in Ohio (Table 1) [4, 37, 38, 40–48]. Lastly, a survey of 108 epilepsy-specialized physicians (neurologist and neurosurgeons) in the United States was included [39].

Overall, 10/13 protocols required acquiring clinical history, while 6/13 required a physical exam (Table 1). All (13/13) mentioned requiring a scalp video/EEG telemetry and structural MRI. The other assessments were required as follows: neuropsychological assessment (11/13), neuropsychiatric assessment (7/13), fMRI (7/13; ancillary for 1/13), Wada test (6/13; ancillary for 2/13), [18] FDG-PET (3/13; ancillary for 9/13), ictal SPECT (3/13; ancillary for 9/13), discussing with patient realistic expectation of outcomes (3/13), MR spectroscopy (2/13), MEG (0/13; ancillary for 6/13), intracranial EEG (0/13; ancillary for 8/13), social care/nursing assessment of support network (2/13), patient management conference (2/13), and occupational evaluation (2/13).

Only protocols by the Spanish Neurosurgical Society and the 2009 consensus conference mentioned assessing the patient’s support network [14, 43]. Other than the 2009 consensus conference, two other protocols mentioned discussing expectations with patients [43, 45]. Lastly, only two protocols mentioned conducing an occupational evaluation and making all surgery decisions in a multidisciplinary management conference [37].

fMRI and the Wada test were required assessments in seven of the protocols (Table 1). [18F]FDG-PET and SPECT were ancillary for all but three articles (where they were required). MEG and intracranial EEG were only mentioned as ancillary. Magnetic resonance (MR) spectroscopy was mentioned as required at two institutes [40, 47].

With regards to the actual indication for selecting patients to begin the investigatory pathway, seven of the articles used a variation of the ILAE definition of refectory epilepsy [37, 38, 42, 43, 45, 46] (Table 2).

**Table 2:** Indications for Beginning Epilepsy Surgery Investigatory Pathway

Citation	Indication for Initiating Workup
UCL Institute of Neurology London, England 2011 [14]	N/A
National Temporal Lobectomy Survey United States 2009 [39]	20% utilize no minimum AED failures to define candidacy
International Neurological Restoration Center Havana, Cuba 2009 [40]	Refractory
University of Florida Gainsville, Florida 2009 [41]	N/A
University of Munich Munich, Germany 2009 [42]	Failure of two or three AED in monotherapy
Spanish Neurosurgical Society Spain 2009 [43]	Seizures regardless of treatment for at least 2 years with two consecutive first-line AEDs and at least one trial of bi-therapy at maximal dosage
R. Madhavan Nayar Center for Comprehensive Epilepsy Care Kerala, India 2010 [37]	Seizures despite treatment with two consecutive first-line antiepileptic medications (AEDs) over 2 years
Cleveland Clinic Cleveland, Ohio 2010 [38]	International League Against Epilepsy definition (a failure of two AED trials as monotherapy or combination)
Barrow Neurological Institute Phoenix, Arizona 2011 [44]	N/A
Oslo University Hospital Oslo, Norway 2012 [45]	International League Against Epilepsy definition (a failure of two AED trials as monotherapy or combination)
Toronto Western Hospital Toronto, Canada 2012 [46]	Seizures despite treatment with two consecutive first-line antiepileptic medications (AEDs) over 2 years
Italian League Against Epilepsy Italy 2013 [4]	Failure of two AED
Gazi University Ankara, Turkey 2014 [47]	Refractory
National Center Hospital for Neurology and Psychiatry Japan 2016 [48]	N/A

## Discussion

Overall, the only assessments in the investigatory pathway required by all protocols were the scalp video/EEG telemetry and structural MRI, while neuropsychological assessment was required in all but two protocols—hence, internationally these three studies appear the most critical for identification of epileptogenic tissue. Otherwise, no two protocols were identical, including the order in conducting the assessments (Table 1). Interestingly, there did not appear to be evident trends based on year of protocol publishing or geography.

In relation to the National Temporal Lobectomy Survey, of the 108 surveyed United States adult epileptologists and neurosurgeons, only 32–36.8% conduct a neuropsychological evaluation [39]. For hemisphere dominance determination, 15.8–32.1% utilized the Wada test exclusively (available at 98% of institutions), while 52–64.7% deemed the test required [39]. fMRI was utilized by 28.6–36.8%, while PET and SPECT demonstrate significant variability in use per provider [39]. In addition, no advanced imaging technologies (i.e., qMRI, 3D-MMI) were utilized amongst any of the protocols, despite the advantage these imaging technologies may make protocol standardization easier and potentially reduce the need of expert examiners, particularly in developing countries. Of note, the survey examines temporal lobectomy cases, hence may not be the most ideal for comparison to the presurgical evaluation in difficult extratemporal case. Additionally, since 2009 many institutions or providers likely have adopted changes to their utilization of invasive assessment in presurgical evaluation.

Some of the variability in test utilization was due to insurance coverage, as some insurance policies did not cover the Wada test or MEG [39]. Therefore, although certain tests may provide valuable insight, socioeconomic restrictions appear to play a role in the investigatory pathway for epilepsy—not just for developing countries. However, cost-effective assessments, such as assessing patient support network and providing realistic expectation of outcomes, were only utilized in few protocols [14, 43, 45]. Moreover, only two protocols mentioned (required or ancillary) using patient management conferences, occupational evaluation, or social care/nursing assessment of patient support networks. Requiring a multidisciplinary approach to surgical evaluation will likely improve patient outcomes, as has been shown in other facets of healthcare [49].

Regarding indications for starting epilepsy surgery workup, patients were selected primarily on the basis of a definition of AED pharmacoresistance by the ILAE. However, roughly 20% of providers in the United States do not use a minimum number of AED failures prior to starting workup [48]. Only the protocol from the Spanish Neurosurgical Society mentions that the semiological intensity of seizures, as well as high seizure frequency and/or social, educational or employment isolation should also be taken into consideration for surgical candidacy; three protocols mentioned at least two-years of AED failure were required [37, 39, 43]. Additionally, with progression in time of protocol publication (from 2009 to 2016), there were no identified trends regarding application of diagnostic techniques or patient work up criteria.

### Limitations

Despite the results, there are several setbacks to the study methods. First, the search criteria was limited to post-2009, and studies included a variety of surgeries/epilepsy types. Regarding the articles themselves, occasionally not all terms were defined (i.e., refractory) and potentially not all aspects of institution protocols were discussed—clinical history and physical exam. Additionally, at each of the centers there was some unavailable data, including the distribution of epilepsy types, diagnostic testing available, and types of operations performed. Lastly, even within institutions there is likely inter-provider variability in patient selection, and the protocols only provide a specific author’s preferences.

## Conclusion

Despite attempts by the ILAE and the 2009 American Epilepsy Society consensus conference to standardize patient selection and investigatory pathway methodologies, there is still significant variation throughout the epilepsy centers globally. According to the majority of protocols, the most useful tests for pre-operative evaluation included scalp video/EEG telemetry, structural MRI, neuropsychological assessment, and fMRI. However, one must note that choice of tests must be tailored to the specific patient and ancillary tests may become required in difficult cases (e.g., extratemporal epilepsy, no clear epileptogenic region boundaries, or when multiple foci are suspected).

Furthermore, we identified several cost-effective evaluations that are under-reported. Particularly those assessments which should be accessible regardless of socioeconomic status, such as neuropsychiatric evaluation, assessment of patient support network via social care/nursing, providing realistic expectation of outcomes, patient management conferences, and occupational evaluation. Incorporating a multidisciplinary approach at all centers could potentially be a cornerstone in patient recovery, as the case in other field of medicine.

If socioeconomic factors play less of a role and as various assessments are improved, there will possibly be a time when a standardized protocol can be applied for investigating epilepsy surgery candidates. Additionally, the evolution of imaging technologies will likely aid the ability to better identify non-overt epileptogenic lesions, which would be particularly useful for centers where neuroimaging experts are not readily available. However, even in developing countries, care must be taken in heavily relying on imaging, as such an approach will potentially be most useful for cases where lesionectomy is the procedure of choice. In more difficult cases, further diagnostic tools (i.e., SEEG, MEG, etc.) and an epileptologist are necessary to ensure patient safety and optimal post-operative outcome, as an oversimplified solution may not be applicable. Overall, we found even amongst expert examiners there is variation throughout epilepsy centers internationally, in selecting candidates and working up patients.

## Supplementary information

**Additional file 1.** Pubmed (MEDLINE) Search Strategy. Embase Ovid Search Strategy. CENTRAL Search Strategy.

## Data Availability

All data generated or analysed during this study are included in this published article [and its supplementary information files].

## Abbreviations

[18F]FDG-PET: Fluorodeoxyglucose F18 positron emission tomography
3D-MMI: Three-dimensional multimodality image
AED: Anti-epileptic drugs
CENTRAL: Cochrane Central Register of Controlled Trails
EEG: Electroencephalogram
fMRI: Functional magnetic resonance imaging
ILAE: International League Against Epilepsy
MEG: Magnetoencephalography
MeSH: Medical subheadings
MRI: Magnetic resonance imaging
qMRI: Quantitative magnetic resonance imaging
SPECT: Single photon emission computed tomography
TLE: Temporal lobe epilepsy
SEEG: Steroelectroencephalography
