# Supplementary material for: Investigatory pathway and principles of patient selection for epilepsy surgery candidates: a systematic review
Source: BMC Neurol. 2020 Mar 17;20:100. doi: 10.1186/s12883-020-01680-w (PMC7079385; doi:10.1186/s12883-020-01680-w)
Supplement: Supplementary file 1 — Additional file 1. Pubmed (MEDLINE) Search Strategy. Embase Ovid Search Strategy. CENTRAL Search Strategy. [file 12883_2020_1680_MOESM1_ESM.docx]

**Additional file 1**

**Pubmed (MEDLINE) Search Strategy**

(((((((((convuls*) OR seizure*) OR epilep*)) AND ((surgi*) OR surger*))) OR epilepsy surgery))) AND ((patient selection) OR ((((selec*) OR selection)) AND patient*))

**Embase Ovid Search Strategy**

(1) exp epilepsy/

(2) epilep*.mp.

(3) exp convulsion.

(4) convuls*.mp.

(5) exp seizure/

(6) seizure*.mp.

(7) exp “seizure, epilepsy and convulsion”/

(8) 1 or 2 or 3 or 4 or 5 or 6 or 7

(9) exp surgery/

(10) surger*.mp.

(11) surgi*.mp.

(12) 9 or 10 or 11

(13) exp patient selection/

(14) (patient adj select*).mp.

(15) 13 or 14

(16) 8 and 12 and 15

Key:

mp = title, abstract, heading word, drug trade name, original title, device manufacturer, drug manufacturer, device trade name, keyword, floating subheading word, candidate term word

**CENTRAL Search Strategy**

1. MeSH descriptor: [Epilepsy] explode all trees
2. Epilep*
3. MeSH descriptor: [Seizure] explode all trees
4. seizure*
5. convuls*
6. #1 or #2 or #3 or #4 or #5
7. surgi*
8. surger*
9. #7 or #8
10. MeSH descriptor: [Patients] explode all trees
11. patient*
12. #10 or #11
13. selec*
14. selection
15. #13 or #14
16. #12 and #15
17. #6 and #9
